# Spontaneous Chalk-Stick Fracture in Ankylosing Spondylitis: A Case Report

**DOI:** 10.31138/mjr.33.3.346

**Published:** 2022-09-30

**Authors:** Chaido Katsimpari, Sofia Koutsoviti, Alexia Mpalanika, Eleni Kalavri, Evangelos Theotikos, Antonis Fanouriakis, Antonia Elezoglou

**Affiliations:** 1 Department of Rheumatology, “Asklepieion” General Hospital, Athens, Greece,; 2 Department of Radiology, “Asklepieion” General Hospital, Athens, Greece

**Keywords:** ankylosing spondylitis, anterior longitudinal ligament rupture, posterior longitudinal ligament rupture, osteoporotic fracture, chalk-stick fracture

## Abstract

Ankylosing spondylitis (AS) is an inflammatory disease affecting mainly the sacroiliac joints and the spine. In long-standing disease, the fused spine of AS patients is susceptible to spinal fractures, even after low impact trauma. We present a 61-year-old man with long-standing AS who presented with anterior and posterior longitudinal ligament rupture and T12 and L1 vertebral endplates fractures (a so called “chalk-stick fracture”) without reporting any prior trauma and discuss relevant issues.

## INTRODUCTION

Ankylosing spondylitis (AS) is a chronic inflammatory disease characterized mainly by spinal ankylosis, inflammation and, ultimately, fusion of the sacroiliac joints and the rest of spine.^1^ Osteoporosis is a common comorbidity in AS and, when superimposed on the fused spine, it results in a higher prevalence of spinal fractures.^2,3^

A type of fractures typically seen in patients with AS are the chalk-stick fractures. These are fractures affecting the vertebrae, the intervertebral disc and the other spinal segments. They are also called carrot stick fractures and typically occur in a fused spine, even following minor trauma.^10^

We herein describe the case of a 61-year-old man with longstanding AS, who experienced a severe chalk-stick fracture following a coughing episode, in the absence of any trauma.

## CASE DESCRIPTION

**Figure 1.**
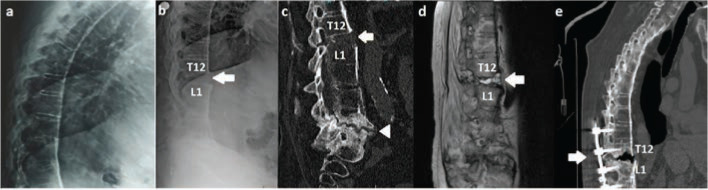
(**a**) Lateral X-ray of thoracolumbar spine showed bamboo spine. (**b**) Lateral X-ray of thoracolumbar spine showed a fracture across the disc and the posterior elements at the T12-L1 level. (**c**) A sagittal-reformatted CT scan of thoracolumbar spine confirmed a horizontally oriented three-column fracture at T12 and L1 level (arrow). An old, inadequately healed chalk stick fracture with sclerotic margins was also seen at L4-L5 level (arrow head). (**d**) Sagittal proton-density MR image showed altered signal intensity of the disc, the vertebral bodies, and the posterior elements at the T12-L1 level. (**e**) A postoperative sagittal-reformatted CT scan of thoracolumbar spine showed extensive fixation with pedicle screws and connective rods.

A 61-year-old obese man (body mass index 30.2 kg/m^2^) with a long-standing history of AS presented to the Emergency Department of our hospital with intense low back pain. Symptoms had started acutely 10 days earlier, following an episode of coughing. No history of fall or any trauma was reported. The patient did not initially seek medical advice, attributing the back pain to his rheumatologic condition. He had been diagnosed with ankylosing spondylitis 40 years ago and had fused sacroiliac joints and a “bamboo” spine (**Figure 1A**). He was receiving treatment with infliximab and methotrexate, as well as bisphosphonates for osteoporosis (zoledronic acid for 3 years). In the past, he had received glucocorticoids for the management of peripheral arthritis, with a total prednisone dose of 1200mg. He was a heavy smoker with more than 50 pack-years. He had also undergone left knee replacement surgery and a cervical spine fusion surgery 40 and 10 years ago, respectively.

On clinical examination in our hospital, the patient was in distress and immobilised due to the intensity of the back pain. Thorough neurological examination did not reveal focal neurological deficits. Plain radiographs showed widening of the intervertebral space between T12 and L1 vertebrae, owing to possible fractures through the inter-vertebral disc and the posterior elements at the T12-L1 level, in the context of a fused thoracic spine (**Figure 1B**). Further imaging evaluation with lumbar computed tomography (CT) and magnetic resonance imaging (MRI) showed additional posterior longitudinal ligament rupture and a fracture in the endplates of T12 and L1 vertebrae (**Figures 1C,D**), all compatible with a “chalk-stick” fracture (horizontally oriented three-column fracture). An old, inadequately healed chalk stick fracture with sclerotic margins was also seen at L4-L5 level (**Figure 1C**). Due to the presumed spinal instability and location of the fracture, the patient underwent posterior long segment pedicle screw fixation (**Figure 1E**). Six weeks post-operatively, he was started again on treatment with infliximab due to reappearance of his AS symptoms. At last follow up, 12 months post-op, the patient has no neurological sequelae and his AS is in remission following infliximab re-administration.

## DISCUSSION

Spinal fractures are up to four times more common in patients with long-standing AS compared to the general population, because the fused spine is vulnerable to fractures. In combination with osteoporosis, such fractures may occur even after low impact trauma. In our patient, it was interesting that intense coughing alone produced enough shearing forces to cause a spinal fracture, in the absence of any trauma. AS patients also tend to have a higher prevalence of neurologic deficits (up to 67%), when a spinal fracture is finally diagnosed.^6^ Additional risk factors for fracture in this population are older age, higher body mass index, longer smoking duration, longer occiput-to-wall distance, pre-existing spinal lesions, longer disease duration and lower hip BMD.^3,4^ In our case, the patient reported a recent DEXA measurement in another hospital showing osteopenia. Regarding location, fractures are more common in the cervical spine, followed by the thoracic, lumbar and sacrum.^5,6^

Due to frequently pre-existing low back pain and common use of analgesics, AS patients with spinal fractures often delay to seek medical attention; consequently, the diagnosis is commonly delayed, resulting in increased risk of neurological deterioration.^3^ The diagnosis may be missed using conventional x-rays, so CT and/or MRI should be performed at a low threshold, even in the absence of trauma. Treatment may be conservative or surgical, depending on fracture location (cervical versus thoracolumbar).^7^ Recent studies suggest that the surgical approach seems to be more effective for thoracolumbar fractures, resulting in better clinical outcomes, mainly improved neurological status and lower mortality rates.^6,8,9^ The preferable surgical approach is the posterior long segment pedicle screw fixation, as was the chosen approach in our patient.^7^

In conclusion, we present an unusual case of lumbar chalk-stick fracture in a patient with AS without any history of trauma. When a patient with long-standing AS experiences acute aggravation of their back pain, it is important to rule out a vertebral fracture; in such cases the threshold for undergoing CT or MRI imaging should be low, even in the absence of a history of trauma.
